# The probability of diagnostic delays for tuberculosis and its associated risk factors in northwest Iran from 2005 to 2016: a survival analysis using tuberculosis surveillance data

**DOI:** 10.4178/epih.e2022060

**Published:** 2022-07-18

**Authors:** Reza Ebrahimoghli, Hassan Ghobadi, Davoud Adham, Parviz Jangi, Abbas Abbasi-Ghahramanloo, Eslam Moradi-Asl

**Affiliations:** 1 Department of Public Health, School of Health, Ardabil University of Medical Sciences, Ardabil, Iran; 2 Division of Pulmonary, Department of Internal Medicine, School of Medicine, Ardabil University of Medical Sciences, Ardabil, Iran

**Keywords:** Tuberculosis, Delayed diagnosis, Survival analysis, Iran

## Abstract

**OBJECTIVES:**

Early diagnosis is essential for effective tuberculosis (TB) control programs. Therefore, this study examined the risk of delays in TB diagnosis and associated factors in Ardabil Province in northwest Iran from 2005 to 2016.

**METHODS:**

This longitudinal retrospective cohort study was conducted using data obtained from the Iranian National Tuberculosis Control Program at the provincial level between 2005 and 2016. The total delay in diagnosis was defined as the time interval (days) between the onset of symptoms and TB diagnosis. Survival analysis was conducted to analyze the delay in diagnosis. Associated factors were identified using a Cox proportional hazards model.

**RESULTS:**

A total of 1,367 new TB cases were identified. The 12-year median diagnostic delay was 45 days (interquartile range [IQR], 30-87). The annual median diagnostic delay decreased from 68 days (IQR, 33-131) in 2005 to 31 days (IQR, 30-62) in 2016. The probability of a delay in TB diagnosis decreased by 5.0% each year (hazard ratio [HR], 1.05; 95% confidence interval [CI], 1.04 to 1.07). Residence in a non-capital county (HR, 0.83; 95% CI, 0.74 to 0.92) and referral from the private health system (HR, 0.74%; 95% CI, 0.65 to 0.84) were significantly associated with an increased risk of delay in TB diagnosis over the 12-year study period.

**CONCLUSIONS:**

The median delay decreased during the study period. We identified factors associated with a longer delay in TB diagnosis. These findings may be useful for further TB control plans and policies in Iran.

## INTRODUCTION

Tuberculosis (TB) is a communicable disease that is a major cause of morbidity and a leading cause of mortality worldwide. According to a World Health Organization (WHO) report, there were approximately 10.0 million new TB cases and 1.5 million deaths from TB globally in 2020 [1]. Although the global TB mortality rate has declined by 37% since 2000 [2], the disease remains one of the top 15 causes of death worldwide [1]. The illness caused by TB also has far-reaching economic, psychological, and social consequences. According to the WHO Global Tuberculosis Report 2021, 47% of TB patients face catastrophic costs based on data from 23 countries that completed a national survey [1].

This disease is both preventable and curable. According to the WHO Global Tuberculosis Report 2021, 86% of new and relapse TB cases registered in 2019 were successfully treated [1]. Therefore, the early diagnosis of TB cases and effective treatment are the keystones of global TB control responses [3,4]. However, delayed diagnosis and treatment of TB cases have been identified as the main obstacles to TB control programs in many countries [5,6]. Delays in the diagnosis and treatment of TB cases can spread the disease within communities, can increase its severity, and are associated with a higher risk of mortality [7,8]. For example, an untreated TB patient can infect, on average, 10-20 people annually [5].

In Iran, TB poses a major health problem and has been intensified by a wide range of delays in overall TB treatment. According to a region-based WHO report, the average delay in diagnosis from the onset of the first symptoms to treatment in Iran is 127 days. Most of this time is spent on diagnosis processing [9]. Although Iran is classified as an upper-middle-income country with a developed healthcare system [10], it borders multiple countries with the highest TB burdens in the region. Pakistan, Afghanistan, and Iraq alone contribute to 80.0% of the regional TB burden [1]. This proximity poses a significant challenge for the Iranian national TB responses [11]. Therefore, it is very important to reduce delays in TB diagnosis and treatment in Iran.

To plan and improve national TB response programs, information on delays in TB diagnosis and associated risk factors is just as important for policy-makers as it is for TB patients and their families. Although there have been multiple studies on delays in TB diagnosis [5,7,9,12,13], analyses and explanations about potential delays can differ based on various factors including local health infrastructure and regional epidemiological characteristics related to TB. In addition, the issue has not been sufficiently investigated in Iran. Furthermore, most existing studies used the delay in TB diagnosis or treatment as a dummy variable and investigated the associated risk factors using classic binary regression models. However, questions have been raised about the appropriateness of classic binary regression analysis, including logistic regression models, when the research question involves the length of time until the endpoint occurs, such as when estimating the median time to diagnose TB cases [14]. This is especially true for data on the time to diagnose TB since there is not an accepted particular “time interval” for delays in TB diagnosis. Therefore, this study aimed to examine delays in TB diagnosis and investigate factors associated with these delays in northwest Iran using a survival analysis approach.

## MATERIALS AND METHODS

### Study design and setting

This was a retrospective cohort study that included all patients diagnosed with TB in Ardabil Province from 2005 to 2016. Ardabil is located in northwest Iran. It covers an area of 17,953 km^2^ (about 1.1% of the whole area of Iran) and is located on an open plain 1.338 m above sea level. According to the latest national census in 2017, Ardabil Province has a population of more than 1.27 million people. It is divided into 9 counties, including Ardabil (the capital county), Bilasavar, Germi, Khakhal, Kowsar, Meshkinshahr, Namin, Nir, and Parsabad.

### Data source

For this study, data at the provincial level from 2005 to 2016 were obtained from the Iranian National Tuberculosis Control Program. In the Iranian TB surveillance system, patients who are suspected of having TB are referred from all forms of health/treatment systems (public or private) to TB diagnosis and treatment centers. In this surveillance system, symptoms that suggest TB are defined according to WHO criteria (including cough, weight loss, night sweats, and fever) [15].

Currently, there are more than 500 free TB diagnostic and treatment centers in the country with at least 1 cultivation center in each province. If TB is confirmed in suspected patients, it is reported by physicians or other healthcare personnel by registering it using the national online platform via any record-keeping system. Next, the reported data are entered into a TB registration software system via double-entry and then transferred to the province level. After a review at the provincial level, data are sent to the national center for disease control [16]. TB diagnoses are made by qualified general physicians and laboratory experts under the careful supervision of an infectious disease subspecialist (referred to as a TB focal point). TB diagnosis and treatment are based on national TB guidelines.

Data collection was performed continuously throughout the full study period and there were no substantial changes in the TB surveillance system or collected data over time.

### Primary outcome and covariate

The TB diagnostic delay was defined as the number of days from the reported onset of symptoms to the date of TB treatment. We did not include TB treatment delays since it is standard practice in Iran to begin anti-TB therapy immediately following diagnosis. Other covariates that were abstracted from the TB surveillance database included age, gender, county of residence (capital or non-capital), area of residence (rural or urban), referring health facility type (public or private), and type of TB (pulmonary or extra-pulmonary).

### Statistical analysis

Descriptive statistics were used to summarize the data in terms of proportion, median, and interquartile range (IQR). Survival analysis refers to an assortment of statistical methods for data analysis for which the target outcome variable is the time until an event occurs [17]. An example of survival time is the interval between the onset of disease and recurrence or death. In analyzing TB diagnostic delays, survival analysis can be applied with the following adaptations: (1) the TB diagnosis can be defined as an event, and (2) survival time can be defined as the time interval between the onset of symptoms and the TB diagnosis. We applied the Kaplan-Meier method, which is a non-parametric estimator of the survival function, as a function of time to estimate the probability of TB diagnosis as the time from the onset of symptoms increased.

To examine the potential effect of various available variables on the probability of TB diagnosis since the onset of symptoms, we performed Cox proportional hazard regression analysis. First, we performed univariable Cox regression analyses and then included all variables with at least a marginal association (p≤ 0.1) in the multivariable model.

For this survival analysis, the outcome of interest was defined as “being diagnosed,” and the time delay was defined as the time interval (days) from the onset of TB symptoms to being diagnosed. Being diagnosed after symptoms onset has been considered as the main event in this survival analysis. Therefore, factors with hazard ratios (HRs) of less than 1.0 were considered to be risk factors associated with an increased risk of delay in TB diagnosis. A 2-sided p-value of < 0.05 was considered to indicate statistical significance for all analyses. Data analysis was performed using Stata version 16.0 (StataCorp., College Station, Texas, USA).

### Ethics statement

This study was approved by the Ethical Regional Committee of Ardabil University of Medical Sciences and Health Services, Ardabil, Iran (Approval ID: IR.ARUMS.REC.1398.135). All experiments were performed in accordance with the relevant guidelines and regulations of the Ministry of Health and Medical Education.

## RESULTS

During the 12 years from 2005 to 2016, a total of 1,619 TB cases were registered in the province-level TB surveillance database, 90 of which were recurrent TB cases and thus excluded from the study. Among the remaining 1,529 TB patients, 162 patients had missing data values for the date of symptom onset. Therefore, a total of 1,367 patients newly diagnosed with TB were included in this study.

Table 1 shows a summary of the baseline characteristics of the included patients. There were 788 (57.6%) pulmonary cases and 579 extra-pulmonary cases (42.4%). Most of the patients were women (53.3%), with a women-to-men ratio of 1.13. The mean± standard deviation age of the participants was 43.7± 19.6 years (median, 42; IQR, 27-59), and a majority were from urban areas (n= 861, 63.0%).

### The pattern of delay in tuberculosis diagnosis

The 12-year median diagnostic delay was 45 days (IQR, 30-87). A total of 942 patients had a prolonged diagnostic delay (>30 days). The delay in diagnosis exceeded 60 days for 547 of the patients (40.0%). The longest diagnostic delay was 917 days, and the shortest amount of time from the onset of symptoms to diagnosis with TB was 0 days.

### Trend

**Figure 1.**
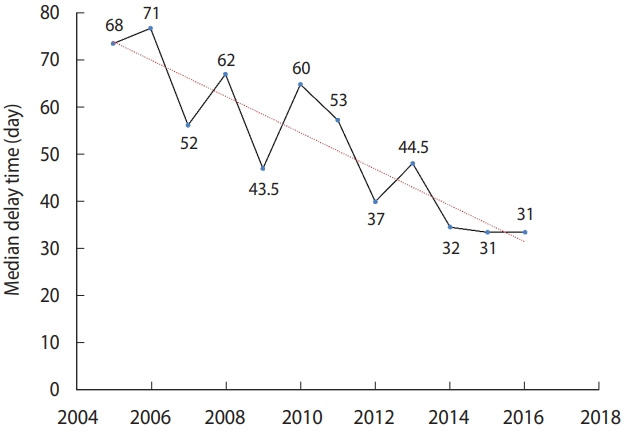
Median delays in tuberculosis diagnosis from 2005 to 2016 in Ardabil Province, northwest Iran.

The median delay until TB diagnosis decreased during the study period. Within the first year of the study, the median delay was 68 days (IQR, 33-131), which dropped to 31 days (IQR, 30-62) in the last year of the study period. The trend in the delay is shown in Figure 1. In the linear regression model, the delay decreased by a median of 5.0% annually.

**Figure 2.**
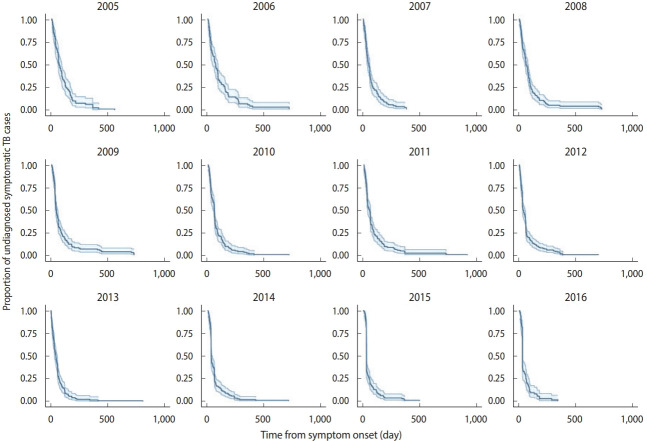
Survival function estimated using the Kaplan-Meier method including 95% confidence intervals. The graph shows the number of patients at risk of delayed tuberculosis (TB) diagnosis at different time points in Ardabil Province, northwest Iran.

Figure 2 shows the survival curves of TB diagnosis over time obtained using the Kaplan-Meier estimator. Over time, the survival curves became more skewed to the left, which indicates fewer delays in recent years compared to early in the study period.

### Diagnosis delays in smear-positive tuberculosis patients

The results of smear examinations were available for a total of 817 patients, 534 of which were smear-positive (65.3%). The median diagnostic delay for these new smear-positive TB patients was 43 days for the 12-year observation period (IQR, 27-85). The annual median diagnostic delay for smear-positive TB cases decreased from 97 days (IQR, 50-125) in 2005 to 41 days (IQR, 22-36) in 2016.

### Factors associated with a longer delay in tuberculosis diagnosis

The multivariate Cox proportional hazards regression analysis (Table 2) showed that patients living in non-capital counties experienced longer delays in diagnosis than patients living in the capital county (adjusted hazard ratio [aHR], 0.83; 95% confidence interval [CI], 0.74 to 0.92; p= 0.001). In the same model, patients utilizing private health systems had a significantly increased risk of delayed TB diagnoses compared to those referred by the public health system (aHR, 0.74; 95% CI, 0.65 to 0.84; p< 0.001). The analysis also showed that patients with extrapulmonary TB experienced longer diagnostic delays compared to those with pulmonary TB (aHR, 0.83; 95% CI, 0.74 to 0.93; p= 0.002). There were no significant differences in the length of TB diagnostic delays according to gender, age group, and area of residence (rural or urban).

## DISCUSSION

Ending the TB epidemic is considered a common aim to be achieved in all countries by 2030 according to the newly adopted Sustainable Development Goals [18]. Given this initiative, early diagnosis and prompt initiation of treatment are integral components of all TB control programs worldwide. In this study, we investigated delays in TB diagnosis and the associated risk factors in northwest Iran.

In this study, the median delay in TB diagnosis was 45 days (IQR, 30-87). This figure matches those observed in earlier studies from other countries such as Egypt (42 days) [14] and India [19]. However, the diagnostic delay estimated in this study was shorter than those reported in multiple other nations, including Afghanistan (200-300 days) [20], Pakistan (91 days) [14], and Somalia (58 days) [14]. These differences can be explained by several factors including patients’ access to quality health services, public awareness of TB, the socioeconomic status of patients, and resource limitations in different settings [21,22].

The overall median diagnostic delay showed a declining trend from 2005 to 2016. The decline in the median TB diagnostic delays each year was also supported by the multivariable cox proportional hazards regression analysis. This trend can be attributed to county-level adaptation to TB reduction strategies and targets with global collaboration [23]. For example, the increased awareness and engagement of communities, civil society organizations, and public and private care providers, together with the establishment of effective TB research centers with quicker and more affordable access to TB diagnosis and treatment, have all had substantial impacts on the reduction in TB diagnostic delays over the past decade [24]. Moreover, the observed reduction in TB diagnostic delays is consistent with the decreasing overall trend of TB incidence in Iran. Kiani et al. [25] found that the overall incidence rate of TB per 100,000 population decreased from 13.4 cases (95% CI, 13.1 to 13.7) in 2008 to 10.8 cases (95% CI, 10.65 to 11.11) in 2018.

We found that various individual and contextual factors affected diagnostic delays. For example, we observed that seeking care from the private health system was independently associated with longer delays than seeking care from the public healthcare system. This is a significant finding in Iran where the private healthcare system is a major provider of medical care [26] and, as observed in our study, more than half of diagnosed TB patients initially sought care from private clinics. One issue highlighted by this finding is the failure to engage the full range of private healthcare providers for TB control within the Iranian health system. This issue was addressed in part by the WHO’s Stop TB Strategy, but private health systems are largely uninterested in building partnerships with national TB control programs [27]. Mutual mistrust between the private and public health sectors, a lack of attractive incentives, weak regulation, and poor accountability may act as substantial barriers to meaningful partnership. Moreover, the lack of continuing medical education to maintain, develop, or increase the knowledge, skills, and professional performance of private healthcare providers may contribute to delays observed among patients referred from private healthcare providers [9].

The current study showed that living in non-capital counties was independently associated with increased delays in diagnosis. This finding indicates that diagnostic capacity may not be distributed equally across counties and that those in non-capital counties may have worse access to quality health services. Thus, it is essential to ensure timely access to appropriate diagnostic services and initiate active campaigns within these counties to identify TB cases that incorporate symptom recording and contact screening in case-finding protocols.

Our analysis showed that 42% of TB cases were extra-pulmonary. This result corresponds to the findings of a previous report from the same region [28]. However, the proportion of extra-pulmonary cases was lower in many countries [1]. Sub-notifications reporting extra-pulmonary TB might underestimate the observed rate of extra-pulmonary TB in these countries due to the difficulty of diagnosing extra-pulmonary TB and the lack of access to adequate diagnostic infrastructure. Therefore, a possible explanation for the high proportion of extra-pulmonary TB in this study might be the superior availability of adequate diagnostic facilities and efficient reporting systems in Iran that lead to the identification of more cases [29].

As in other studies [5,12,30], the presence of extra-pulmonary TB increased the probability of a delay in TB diagnosis. This finding is not surprising, since extra-pulmonary TB diagnoses require multiple clinical presentations, making diagnosis difficult. From a public health perspective, extra-pulmonary TB cases have fewer consequences in terms of the spread of infection. However, these cases may pose a diagnostic challenge for healthcare providers and could easily be overlooked.

In addition to established risk factors, our findings highlight a novel risk factor for delays in TB diagnosis.

The findings of the current study do not support the previously reported associations between delayed TB diagnosis and gender [31,32]. A possible explanation for this result may be different gender-based social influences in health decision-making across cultures and countries [33]. Similarly, our analysis was unable to replicate the results of multiple previously published studies showing a significant association between the area of residence (rural or urban) and delays in TB diagnosis [34,35]. These results may be due to differences in health systems. Most patients in rural areas of Iran have primary access to health posts (the first level of the Iranian health system), into which the TB control program has been integrated. Therefore, the health infrastructure of Iran may have reduced potential discrepancies in diagnostic delays between rural and urban dwellers.

The study has several limitations that should be considered when interpreting the results. First, discrepancies between the actual and reported date of the onset of symptoms could have led to an underestimation or over-estimation of the delay. However, to minimize recall bias when reviewing medical records, local calendar dates, national holidays, and religious days were used as benchmarks. In addition, we were unable to categorize treatment delays as either patient-related or health system-related delays due to limited data on patients’ first contact with healthcare providers. Another potential limitation is the retrospective nature of the study and the lack of data on possible undetected TB cases. Finally, our database did not include covariates, such as socioeconomic status, education level, access to care, and symptom profile, which are important cofounders of diagnostic delays.

## Figures and Tables

**Table 1.** Characteristics of newly diagnosed tuberculosis patients in Ardabil Province, northwest Iran (2005-2016)

Characteristics	n (%)
Total	1,367 (100)
Gender	
Men	639 (46.7)
Women	728 (53.3)
Age (yr)	
Mean±standard deviation	43.7±19.6
Median (interquartile range)	42 (27-59)
>50	831 (61.0)
≤50	533 (39.0
County	
Capital county	632 (46.3)
Non-capital county	735 (53.7)
Area of residence	
Urban	861 (63.0)
Rural	506 (37.0)
Health system	
Private health system	756 (55.3)
Public health system	611 (44.7)
Tuberculosis type	
Pulmonary	788 (57.6)
Extra-pulmonary	579 (42.4)

**Table 2.** Factors associated with delays in tuberculosis diagnosis in Ardabil Province, northwest Iran (2005-2016)

Variables	Diagnosis delay (day)	Univariate analysis	p-value	Multivariate analysis	p-value
Gender					
Women	44 (30-89)	1.00 (reference)	-	-	-
Men	46 (28-85)	1.03 (0.92, 1.14)	0.560	-	-
Age (yr)					
>50	41 (27-83)	1.00 (reference)	-	-	-
≤50	52 (30-92)	0.98 (0.87, 1.09)	0.720	-	-
County					
Capital county	31 (30-76)	1.00 (reference)	-	1.00 (reference)	-
Non-capital county	53 (29-93)	0.89 (0.80 , 1.00)	0.050	0.83 (0.74,0.92)	0.001
Area of residence					
Rural	52 (30-82)	1.00 (reference)	-	-	-
Urban	41 (29-92)	1.03 (0.92, 1.15)	0.500	-	-
Health system					
Public health system	31 (29-68)	1.00 (reference)	-	1.00 (reference)	-
Private health system	60 (30-115)	0.69 (0.61, 0.78)	<0.001	0.74 (0.65, 0.84)	<0.001
Tuberculosis type					
Pulmonary	39 (26-79)	1.00 (reference)	-	1.00 (reference)	-
Extra-pulmonary	53 (30-94)	0.81 (0.72, 0.90)	<0.001	0.83 (0.74, 0.93)	0.002
Study year	-	1.05 (1.03, 1.07)	<0.001	1.05 (1.04, 1.07)	<0.001

Values are presented as median (interquartile range) or hazard ratio (95% confidence interval).
